# Integrated transcriptomics and histopathology approach identifies a subset of rejected donor livers with potential suitability for transplantation

**DOI:** 10.1186/s12864-024-10362-7

**Published:** 2024-05-02

**Authors:** Ankita Srivastava, Alexandra Manchel, John Waters, Manju Ambelil, Benjamin K. Barnhart, Jan B. Hoek, Ashesh P. Shah, Rajanikanth Vadigepalli

**Affiliations:** 1 Daniel Baugh Institute for Functional Genomics and Computational Biology, Department of Pathology and Genomic Medicine, Thomas Jefferson University, Philadelphia, PA 19107 USA; 2 Department of Surgery, Thomas Jefferson University Hospital, Jefferson University Hospitals, Philadelphia, PA USA

**Keywords:** Liver transplantation, Human donor liver, RNAseq, Histopathological scoring, Functional enrichment, Artificial intelligence-based image analysis

## Abstract

**Background:**

Liver transplantation is an effective treatment for liver failure. There is a large unmet demand, even as not all donated livers are transplanted. The clinical selection criteria for donor livers based on histopathological evaluation and liver function tests are variable. We integrated transcriptomics and histopathology to characterize donor liver biopsies obtained at the time of organ recovery. We performed RNA sequencing as well as manual and artificial intelligence-based histopathology (10 accepted and 21 rejected for transplantation).

**Results:**

We identified two transcriptomically distinct rejected subsets (termed rejected-1 and rejected-2), where rejected-2 exhibited a near-complete transcriptomic overlap with the accepted livers, suggesting acceptability from a molecular standpoint. Liver metabolic functional genes were similarly upregulated, and extracellular matrix genes were similarly downregulated in the accepted and rejected-2 groups compared to rejected-1. The transcriptomic pattern of the rejected-2 subset was enriched for a gene expression signature of graft success post-transplantation. Serum AST, ALT, and total bilirubin levels showed similar overlapping patterns. Additional histopathological filtering identified cases with borderline scores and extensive molecular overlap with accepted donor livers.

**Conclusions:**

Our integrated approach identified a subset of rejected donor livers that are likely suitable for transplantation, demonstrating the potential to expand the pool of transplantable livers.

**Supplementary Information:**

The online version contains supplementary material available at 10.1186/s12864-024-10362-7.

## Introduction

Liver transplantation is a crucial intervention for acute [1–3] and chronic liver failure [4], addressing a range of liver disease conditions [5–10]. While there has been a significant improvement in post-transplant survival rates, there is an increasing demand for donor livers available for transplants. Recent metrics from the Organ Procurement and Transplantation Network (OPTN) in the United States indicate an 11.18% increase in the number of deceased donors, reaching 8,788 donors compared to the previous year. However, there remains an unmet demand for liver transplantation because not all donated livers are transplanted (7.2% are not transplanted per OPTN [11] ). Additionally, 12,053 recipients were added to the waitlist for liver transplantation in the previous year (as of October 27, 2023 [11] ).

Current clinical selection criteria for donor livers include liver function tests to evaluate the levels of alanine aminotransferase (ALT), aspartate aminotransferase (AST) [12, 13], total bilirubin, blood type compatibility between the donor and recipient [14, 15], absence of viral infections such as Hepatitis and HIV [16, 17], assessment of alcohol and drug-induced liver damage and histopathological evaluation of liver biopsy for steatosis, fibrosis, and necrosis [18, 19]. These comprehensive criteria aid in determining the suitability of donor livers for transplantation [20]. However, variability in the application of the clinical selection criteria between transplant centers results in the rejection of a fraction of donor livers for transplantation [21]. Furthermore, rejected donor livers are highly variable, with a range of differences in several parameters, including tissue steatosis, fibrosis, donor age, and underlying conditions. The wide range of variability among rejected donor livers could potentially lead to the rejection of a fraction of rejected donor livers that might be suitable for transplantation. For instance, livers from donation after circulatory death (DCD) show high reperfusion injury in contrast to livers from donation after brain death (DBD), which reduces graft success post-transplantation [22]. There is variability in the perfusion time between DBD and DCD livers [23], and the difference in the perfusion time influences the downstream functions impacting graft success [24, 25].

To address the variability between transplant centers and within the pool of rejected donor livers, a comprehensive characterization of donor livers beyond histopathology is warranted. Transcriptomics approach provides a global view of the underlying molecular processes and enables deeper insights into the pathophysiological mechanisms of liver function and regeneration. Previous studies have focused on characterizing the transcriptomic profiles of the liver to predict the post-transplantation outcomes, including the effects of ischemia-reperfusion injury [26] and initial graft function [27]. However, to date, no study has focused on the transcriptomic analysis of the donor livers for assessing the similarities and differences between the accepted and rejected livers, which can yield new information for augmenting the current criteria to evaluate the suitability for transplantation.

Here, we applied an integrated molecular and histopathological approach to characterize donor livers that were either accepted or rejected for transplantation. We evaluated the highly variable rejected group for differences and overlap with the accepted donor livers. Our analysis aimed to identify a subset, if any, of rejected donor livers that shares molecular and histopathological features with accepted donor livers.

## Materials and methods

### Ethical approval and sample collection

All research was conducted in accordance with both the Declarations of Helsinki and Istanbul. Informed Consent to Research was obtained by the Gift of Life Foundation to collect liver biopsy samples from the deceased donors. As per the regulations outlined in 45 CFR 46.101 (b) [4], the current study falls under Exemption 4, and does not require additional Institutional Review Board approval. A Certification for Protected Health Information of Decedents for Research was obtained from the Privacy Officer of the Office of University Counsel and Corporate Compliance Department of Thomas Jefferson University Hospital (TJUH). The informed consent was obtained by the Gift of Life Foundation from the deceased donor`s next of kin to collect liver biopsy samples for research purposes. Clinical information regarding patient demographics, including age, sex, race, body mass index, AST, ALT, and total bilirubin levels was collected (Table 1). Tissue samples were categorized into two groups: (1) the accepted group (*n* = 10), corresponding to donor livers accepted for transplantation by the TJUH Liver Transplant Center, and (2) the rejected group (*n* = 22), consisting of donor livers rejected for transplantation by multiple transplant centers (Table S1). Liver wedge biopsy tissue samples were obtained at the time of organ recovery. A portion of the tissue was flash-frozen and another was fixed in paraformaldehyde.

### RNA extraction and sequencing

Total RNA was extracted from flash-frozen liver tissue samples using the miRNeasy Mini Kit (217,004; Qiagen, Hilden, Germany), and the extracted RNA was stored at -80 °C. The purity and quality of the RNA were assessed using a Bioanalyzer system (Agilent Technologies, California, United States). RNA sequencing was performed at Thomas Jefferson University Genomics Core Facility. Libraries were prepared using the Illumina Stranded Total RNA Prep with Ribo-Zero (20,040,525; Illumina, California, United States) and sequenced on a NovaSeq 6000 platform (Illumina). Paired-end sequencing was performed with read lengths of 2 × 100 bp, and each sample had a sequencing depth of at least 50 million reads. Fastq generation was performed using the Basespace cloud-based analysis platform (Illumina).

### Data processing and quality control

We used the nf-core RNA-seq pipeline (version 3.7; doi: 10.5281/zenodo.1400710) within the Nextflow framework (version 22.04.0) [28]. This pipeline facilitated fastq preprocessing, quality assessment, transcriptome alignment using STAR aligner [29], and transcript quantification using salmon [30]. Alignments were performed against the GRCh38 genome using Gencode v40/ENSEMBL gene annotations [31]. One sample was an outlier with total gene counts less than two standard deviations below the mean across all 32 samples and was excluded from the analysis. The RNAseq data files have been deposited in the NCBI Gene Expression Omnibus under the accession number GSE243887. The normalized gene expression values are available in Table S2.

### Differential gene expression analysis

The raw gene counts were normalized to counts per million (CPM), and genes with the sum of expression values across all samples less than 10 CPM were filtered out. The DESeq2 package (version 1.38.2) was used for differential gene expression analysis [32]. The raw filtered count data was normalized using the variance stabilizing transformation (*vst*) function from the DESeq2 package. Genes with two-fold up or down-regulation and p_adj_ < 0.01 were selected for downstream functional pathway enrichment analysis. Dimension reduction was performed using the *umap* R package [33]. K-means clustering with k = 2 was performed on the *vst* normalized data for accepted and rejected groups.

### Estimating sensitivity of the statistical test

We performed a power analysis using the *RNASeqPower* package in R to assess the statistical confidence of our study based on the study sample size [34]. We estimated sensitivity across various comparison groups: accepted vs. rejected, accepted vs. rejected-1, rejected-2 vs. rejected-1, and accepted vs. rejected-2 groups.

### Pathway enrichment and network analysis

Functional annotation and gene ontology analysis were conducted using the DAVID tool version 2021 [35]. Protein-protein interaction (PPI) networks were constructed using the Cytoscape String App [36], and highly interconnected regions within the network were identified using the Molecular Complex Detection (MCODE) app [37].

### Comparison with transcriptomics of graft failure post-transplantation

We used the results from an earlier study that developed a set of classifier genes (78 genes) that are differentially regulated in livers displaying initial poor graft function (IPGF) post-perfusion and transplantation [27]. There were 69 downregulated and 7 upregulated genes in the non-IPGF vs. IPGF group (Table 5 of Defamie et al., 2008 [27]). We performed Gene Set Enrichment Analysis using *fgsea* package in R to evaluate whether the non-IPGF downregulated genes were enriched in the present differential gene expression comparisons (rejected-2 vs. rejected-1 and accepted vs. rejected-1). Differentially expressed genes in our study were ranked in decreasing order of log2 fold change. Enrichment scores were calculated based on the distribution of non-IPGF classifier genes in the rank-ordered differentially expressed gene list. Since the non-IPGF upregulated genes had only 4 genes, it was not statistically meaningful to evaluate enrichment.

### Histopathological staining

We preserved 26 of 31 donor liver biopsies for independent histopathological evaluation (*n* = 7 accepted, *n* = 7 rejected-1, *n* = 12 rejected-2). Liver biopsy tissue samples (∼2 mm diameter) were locked in Tissue Embedding Cassettes and fixed in 4% paraformaldehyde (PFA) (Electron Microscopy Sciences, Hatfield, PA, United States) for 24 h on a shaker. The cassettes with the biopsy samples were washed three times in distilled water and then placed in 70% ethanol (Decon Labs, Pennsylvania, United States) at 4 °C until paraffin embedding. Histopathology processing was performed by the Pathology Core at Thomas Jefferson University. The sections were deparaffinized in xylene (534,056; Sigma Aldrich, Massachusetts, United States) and rehydrated in a series of decreasing ethanol concentrations, followed by distilled water. Serial sections were stained with Hematoxylin & Eosin (H&E) and Masson’s Trichrome.

H&E staining: Liver tissue sections were first incubated in Haematoxylin (SL100; StatLab, Texas, United States) for 2 min at room temperature and then washed under running cold water for 1 min. The sections were next incubated in acid water (SL103; StatLab) for 1 min, washed under running cold water for 1 min, and then incubated in Bluing Reagent (SL102; StatLab) for 1 min, followed by two washes in deionized water. Slides were then incubated in 95% Alcohol for 1 min, followed by incubation in Eosin (SL104; StatLab) for 2 min.

Trichrome staining: The sections were incubated in Mordant in Bouin’s Fixative (s129; Poly Scientific R&D Corp, New York, United States) for 1 h at room temperature. The sections were first gently rinsed in running tap water for 10–15 min and then rinsed once in distilled water. Next, the sections were incubated in Weigert’s Iron Hematoxylin Working Solution (s216b; Poly Scientific R&D Corp) for 10 min, gently washed in warm water for 10–15 min and then rinsed in distilled water (10 dips) for three changes of distilled water. The sections were incubated in Biebrich Scarlet – Acid Fuchsin (s125; Poly Scientific R&D Corp) for 5 min and rinsed twice in distilled water. The sections were then differentiated in Phosphotungstic – Phosphomolybdic Acid (s225; Poly Scientific R&D Corp.) for 12 min and then placed in Aniline Blue Solution (s116; Poly Scientific R&D Corp) for 20 min. The sections were rinsed twice with distilled water for 15 s each. Next, the sections were incubated in 1% Aqueous Acetic Acid (s100; Poly Scientific R&D Corp) for 3 min. Lastly, the sections were dehydrated in increasing ethanol concentrations, followed by xylene, and then coverslipped.

### Histopathological scoring for liver steatosis, fibrosis, and necrosis

Whole slide images (WSIs) were acquired at ×20 magnification using an Aperio ImageScope digital slide scanner (Leica Biosystems, Wetzlar, Germany). Histopathological evaluation of steatosis, fibrosis, necrosis, and hepatocyte ballooning was performed by a gastrointestinal pathologist. In parallel, the percentage of steatosis and tissue collagen were assessed using pre-trained AI-based image analysis applications in Visiopharm software (Visiopharm Corporation, Colorado, United States). The areas of collagenous and steatotic tissues were divided by the total tissue area to calculate the respective percentages shown in Table 2.

## Results

### Identification of molecularly defined human donor liver subsets

**Fig. 1:**
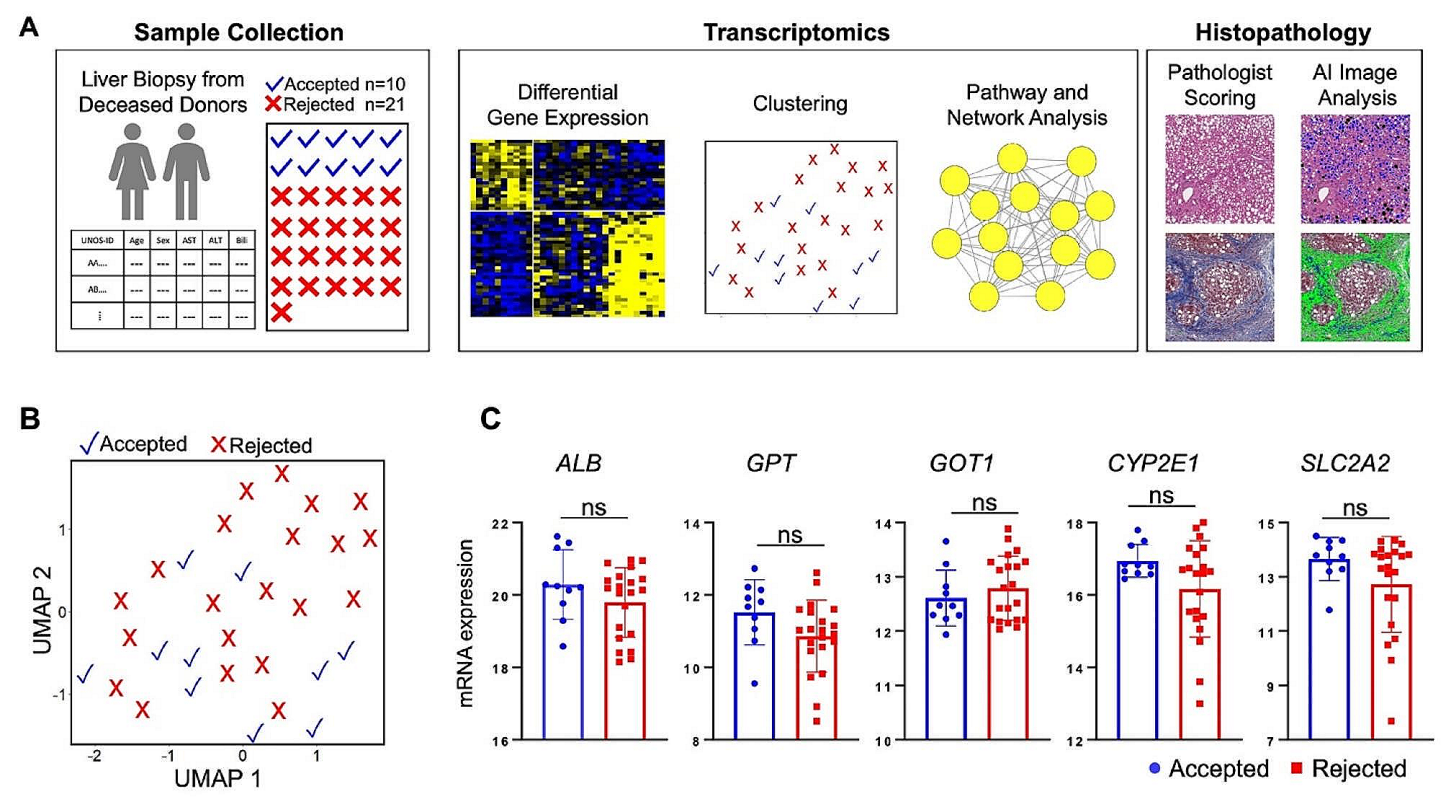
A comprehensive approach integrating transcriptomics and histopathology to identify the donor livers potentially suitable for transplantation. (**A**) Overview of the multi-modal data acquisition and analysis workflow. (**B**) Dimension reduction analysis of transcriptomic data using Uniform Manifold Approximation and Projection **(**UMAP) illustrating the molecular overlap between accepted and rejected donor livers. (**C**) mRNA expression levels of select liver genes in the accepted (*n* = 10) and rejected (*n* = 21) groups. The statistical significance was determined using the DESeq2 package in R. Error bars: mean ± standard deviation, ns: p_adj_>0.05

We applied an integrated approach, including evaluation of clinical data, histopathology, and transcriptomics, to identify a subset of rejected donor livers that may be suitable for transplantation (Fig. 1A). We obtained liver biopsies from deceased donors at the time of organ recovery, either accepted or rejected for liver transplantation. Information on donor demographics and standard serological tests is provided in Table 1. We performed RNA sequencing of donor liver biopsies to characterize the global gene expression profiles and enriched functional pathways. In parallel, a histopathological evaluation was performed to assess steatosis, fibrosis and necrosis.

**Table 1:** Characteristics of deceased donors include sex, age, race, cause of death, BMI, serum ALT, AST, and total bilirubin levels. The samples identified as potentially transplantable from the integrated molecular and histopathological analysis are highlighted in bold text

UNOS-ID	Group	Sex	Age	Race	Cause of Death	BMI	ALT (U/L)	AST (U/L)	Total Bili (mg/dl)
AIWL117	Accepted	Female	35	Caucasian	DBD	31.2	285 − 52	326 − 94	0.2–0.6
AHHC458	Accepted	Female	30	Hispanic	DBD	30.5	39–112	25–256	0.5–2.1
AHHQ205	Accepted	Male	23	Caucasian	DBD	23	223 − 32	169 − 24	0.3 − 0.1
AIBQ043	Accepted	Female	44	African American	DCD	26.5	104–106	159 − 93	0.5–1.8
AIBF419	Accepted	Male	59	Caucasian	DBD	49.6	12–78	15–37	0.2-1
AJCN178	Accepted	Male	56	Caucasian	DCD	26.3	107–139	171–447	0.2–0.5
AJCT312	Accepted	Male	81	Caucasian	DBD	26.8	8–8	13 − 11	1.3–1.3
AIGS266	Accepted	Female	35	Caucasian	DBD	29.5	25 − 23	52 − 49	1.3–0.9
AIDY465	Accepted	Female	48	Caucasian	DBD	27.4	82–100	114 − 58	0.8–0.9
AHLX028	Accepted	Female	41	Caucasian	DBD	43.5	89 − 81	64 − 44	0.2–0.4
AHH5160	Rejected-1	Male	51	Caucasian	DCD	22.6	181 − 114	425 − 223	1.5–7.4
AHIE299	Rejected-1	Male	23	Caucasian	DBD	24.5	1419–5000	1129–4930	0.6–6.5
AHHH143	Rejected-1	Male	49	Caucasian	DCD	37.5	171 − 103	262–514	1-2.6
AHG2102	Rejected-1	Male	47	Caucasian	DBD	40	28 − 24	114 − 83	2.3–4.1
AHKL087	Rejected-1	Male	57	Caucasian	DCD	27.3	730–1013	1144–2496	0.6-3.0
AHIR183	Rejected-1	Male	63	Caucasian	DBD	32	221 − 40	701 − 479	0.4–0.4
AHGW406	Rejected-1	Female	57	Caucasian	DCD	36.3	249–3914	235–3327	0.3–0.7
AIAC389	Rejected-1	Female	68	Caucasian	DBD	18.5	136–1072	200–2116	2.5–4.5
AIAJ296	Rejected-1	Male	42	Caucasian	DCD	50.9	252–997	263–1565	1.2–1.7
AHK2146	Rejected-2	Male	49	Hispanic	DCD	43.6	72–90	64–75	0.2–0.3
AHL5250	Rejected-2	Male	58	Caucasian	DBD	36.1	109 − 83	108 − 100	0.5 − 0.4
AHKN132	Rejected-2	Male	52	Caucasian	DBD	26.2	16 − 11	22–22	0.8 − 0.7
AHJQ339	Rejected-2	Female	31	Hispanic	DCD	28.6	31 − 15	21 − 13	0.7–0.8
**AHGI142**	**Rejected-2**	**Female**	**73**	**Caucasian**	**DCD**	**25**	**48–70**	**57–200**	**0.3–0.6**
AHGO418	Rejected-2	Female	66	Caucasian	DBD	32.9	20–828	100–1189	0.4–0.6
**AHJB400**	**Rejected-2**	**Male**	**41**	**African American**	**DBD**	**35.8**	**87-3111**	**77-2357**	**0.2–2.7**
**AHII090**	**Rejected-2**	**Male**	**67**	**African American**	**DCD**	**47.4**	**12 to 24**	**27–42**	**0.4–0.9**
AHLH202	Rejected-2	Female	62	Caucasian	DCD	53	38 − 37	74 − 45	0.2–0.4
**AHLJ351**	**Rejected-2**	**Male**	**51**	**Caucasian**	**DCD**	**32.7**	**579 − 64**	**630 − 16**	**0.6–0.6**
AHKC275	Rejected-2	Female	45	Caucasian	DBD	36.6	31–46	42–139	0.6 − 0.5
**AHCE041**	**Rejected-2**	**Female**	**59**	**Caucasian**	**DCD**	**41.8**	**44–46**	**44–105**	**0.2–0.3**

To characterize the molecular differences between the accepted and rejected donor livers, as well as the variability within the rejected group, we performed dimension reduction using Uniform Manifold Approximation and Projection (UMAP) on the normalized transcriptomics data on the expression of 18,033 genes. The accepted and rejected groups were not distinctly separated within the broader molecular heterogeneity of the donor livers (Fig. 1B). We examined the expression of select genes critical to liver function and homeostasis, including albumin (*ALB)*, glutamate pyruvate transaminase gene (*GPT*) encoding ALT, glutamic-oxaloacetic transaminase gene (*GOT*) encoding AST, cytochrome P450 Family 2 Subfamily E Member 1 (*CYP2E1)*, and solute Carrier Family 2 Member 2 *(SLC2A2)*. We observed an overlapping gene expression between the accepted and rejected groups (Fig. 1C). We found a high variability in gene expression in the rejected group, with a fraction of the rejected donor livers and accepted livers showing similar expression levels of key genes (Fig. 1C).

### Molecular profiling indicates extensive gene expression overlap between accepted and rejected donor livers

**Fig. 2:**
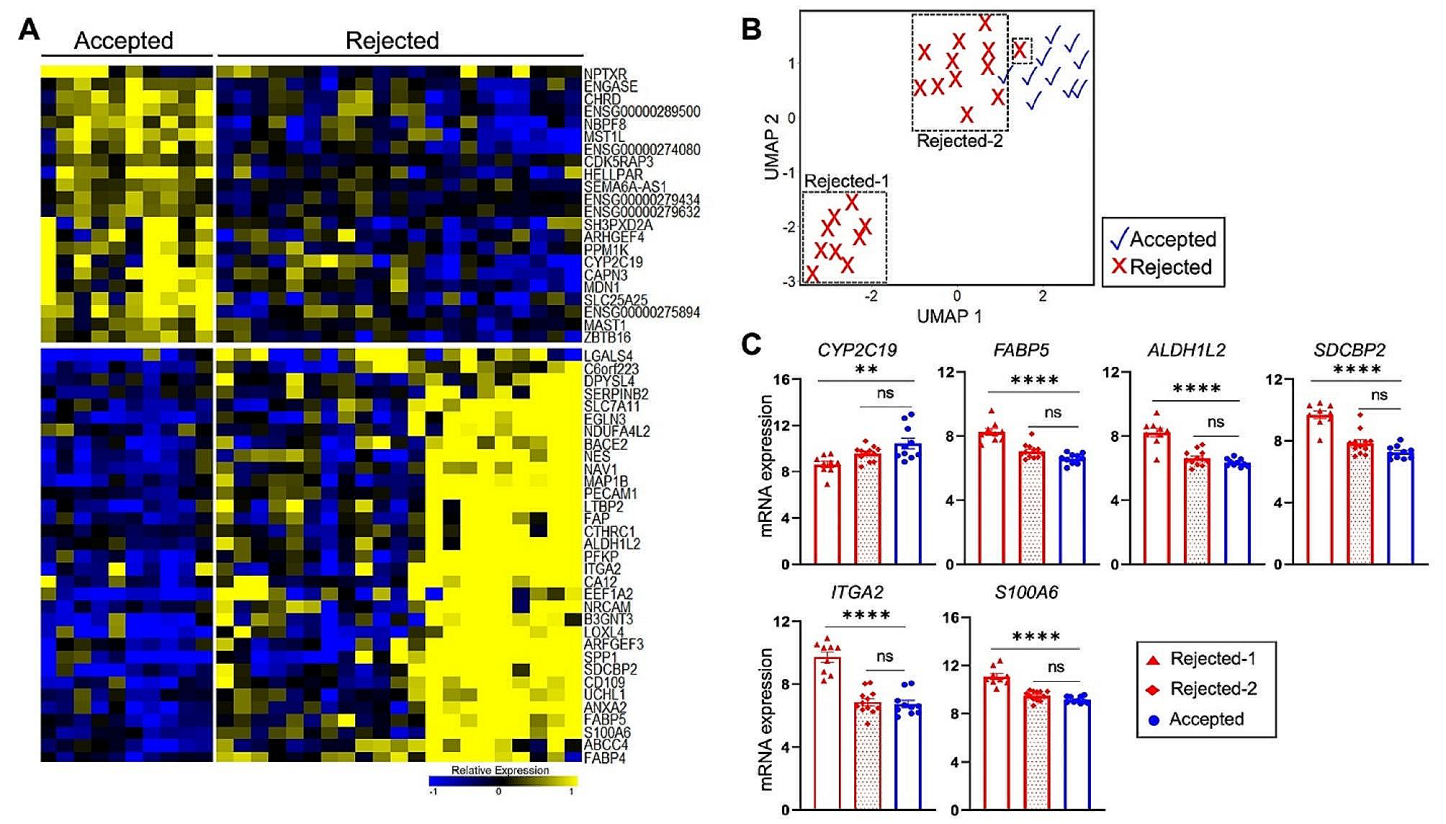
Distinguishable subsets within the molecularly heterogeneous rejected group. (**A**) Heatmap displaying the expression levels of the significant differentially expressed genes (padj < 0.01) between accepted and rejected groups. **B:** UMAP based on 55 differentially expressed genes followed by k-means clustering with k = 2. The two distinguishable subsets of the rejected group (rejected-1 and rejected-2) are indicated. **C:** mRNA expression levels of select genes from panel A to illustrate the subsets within the variable rejected group. Statistical significance was calculated using one-way ANOVA Error bars: mean ± standard error of the mean, one-way ANOVA ****p_adj_<0.0001,**p_adj_<0.01, n.s. p_adj_>0.05

Differential gene expression analysis between the accepted and rejected liver donor groups yielded only 55 of the 18,033 genes as statistically significant (p_adj_<0.01, estimated sensitivity of 87.95%, Fig. 2A). The corresponding list of differential gene expression fold changes is included in Supplementary Table S3. Visualization of the expression profiles of these 55 genes suggests that a subset of rejected donor livers exhibit similar gene expression profiles to that of the accepted group. Dimension reduction analysis and classification based on these 55 genes identified two molecularly distinguishable subsets within rejected donor livers (rejected-1 and rejected-2 groups hereon) (Fig. 2B). Of the 21 rejected donor livers, 9 were classified as rejected-1 and 12 as rejected-2. The rejected-2 subset clustered closely with the accepted group, while the rejected-1 subset was further away from the accepted group. Analysis of the selected genes involved in drug metabolism (*CYP2C19*), fatty acid metabolism (*FABP5, ALDH1L2*), cell adhesion (*SDCBP2*), cytoskeletal organization (*ITGA2*), inflammation, and hepatic stellate cell activation (*S100A6*) illustrated the extensive molecular overlap between the accepted and rejected-2 groups (Fig. 2C).

### A subset of rejected donor livers share broad transcriptomic features with the accepted group

**Fig. 3:**
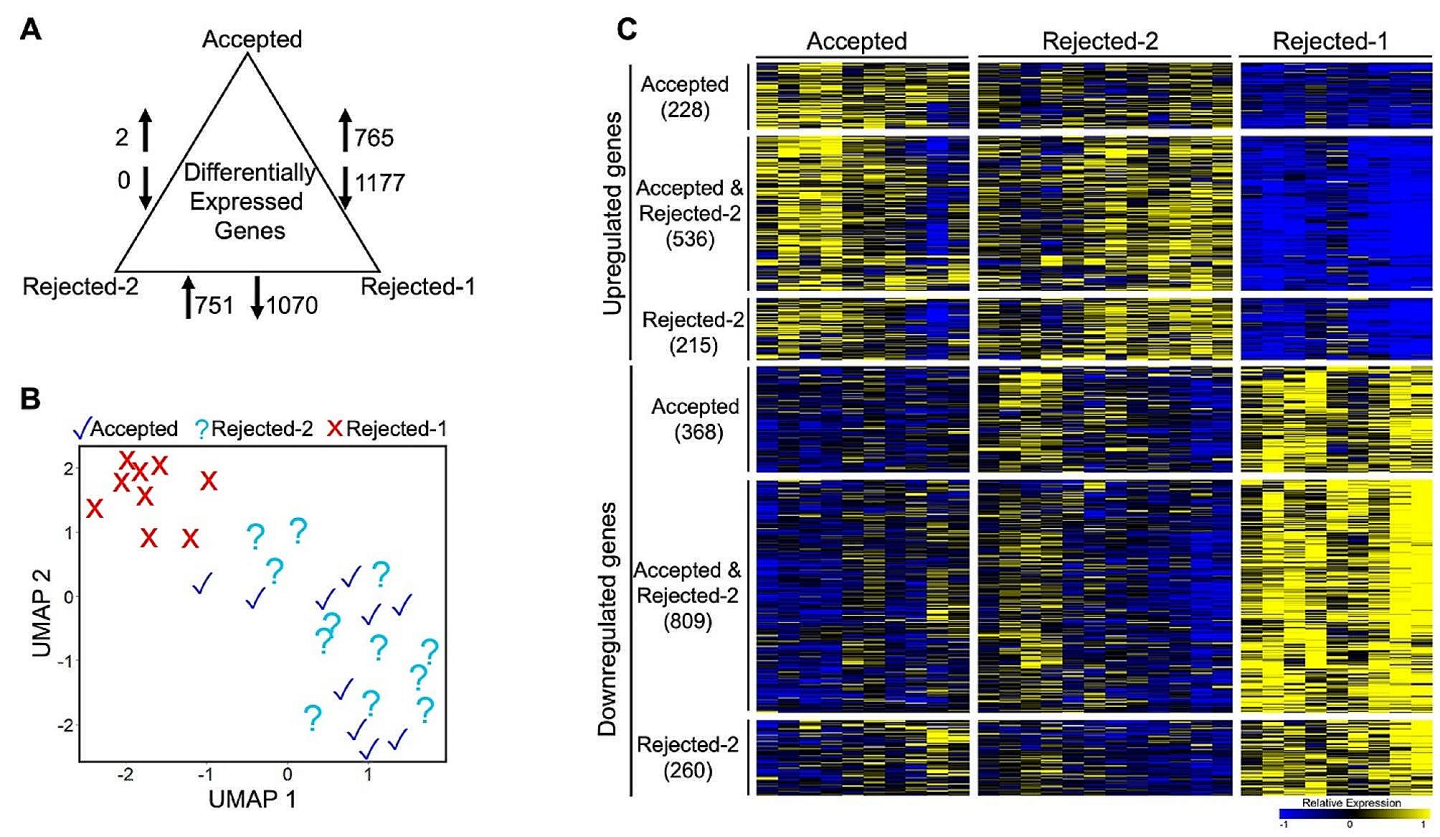
Extensive transcriptomic overlap between a subset of rejected donor livers and the accepted group. (**A**) Differential gene expression analysis comparing the Accepted, Rejected-1, and Rejected-2 groups (p_adj_ <0.01, fold change > 2). (**B**) UMAP analysis of 2418 differentially expressed genes in either accepted or rejected-2 groups identified two distinct clusters comprising accepted and rejected-2 as one cluster and rejected-1 as a separate cluster. (**C**) Heatmap displaying the expression profiles of statistically significant up and downregulated genes distributed between the accepted and rejected groups (p_adj_ <0.01, fold change > 2). The expression values are median-centered for each gene to illustrate the differential gene expression profiles across the three sample groups

We determined the significantly differentially expressed genes (DEGs) between three groups: accepted vs. rejected-1, accepted vs. rejected-2, and rejected-2 vs. rejected-1 (padj < 0.01, two-fold up or down). Our analysis revealed a total of 1942 DEGs between the accepted and rejected-1 groups (765 upregulated genes and 1177 downregulated genes, estimated sensitivity of 79.62%), and 1821 DEGs between the rejected-2 and rejected-1 groups (751 upregulated genes and 1070 downregulated genes, estimated sensitivity of 83.42%, Fig. 3A). There were 2 DEGs between the accepted and rejected-2 donor livers (2 upregulated genes and no downregulated genes, estimated sensitivity 93.46%), indicating extensive and transcriptome-wide molecular overlap between the accepted and the rejected-2 subset. The corresponding lists of differential gene expression fold changes for the three pairwise comparisons are included in Supplementary Table S4. Dimension reduction analysis based on the DEGs in either the accepted and rejected-2 groups compared to the rejected-1 group revealed the rejected-1 group was separate from the other two groups (accepted and rejected-1). There was an extensive overlap between the accepted and the rejected 2 groups (Fig. 3B). A comparison of the differential gene expression profiles in the accepted and rejected-2 groups suggests that the majority of the genes show similar expression patterns (Fig. 3C). The two rejected subsets had similar proportions of DBD and DCD cases (Table 1 - rejected-2: 58% DCD; rejected-1: 55% DCD). Overall, our findings suggest that the rejected-2 subset within the highly variable rejected group shared many molecular features with the accepted donor livers, indicating potential similarities in their underlying molecular states and, by implication, in their functional capacities in multiple pathways critical to liver pathophysiology.

### Shared molecular features between accepted and a subset of rejected donor livers contribute to metabolic functions and extracellular matrix organization

**Fig. 4:**
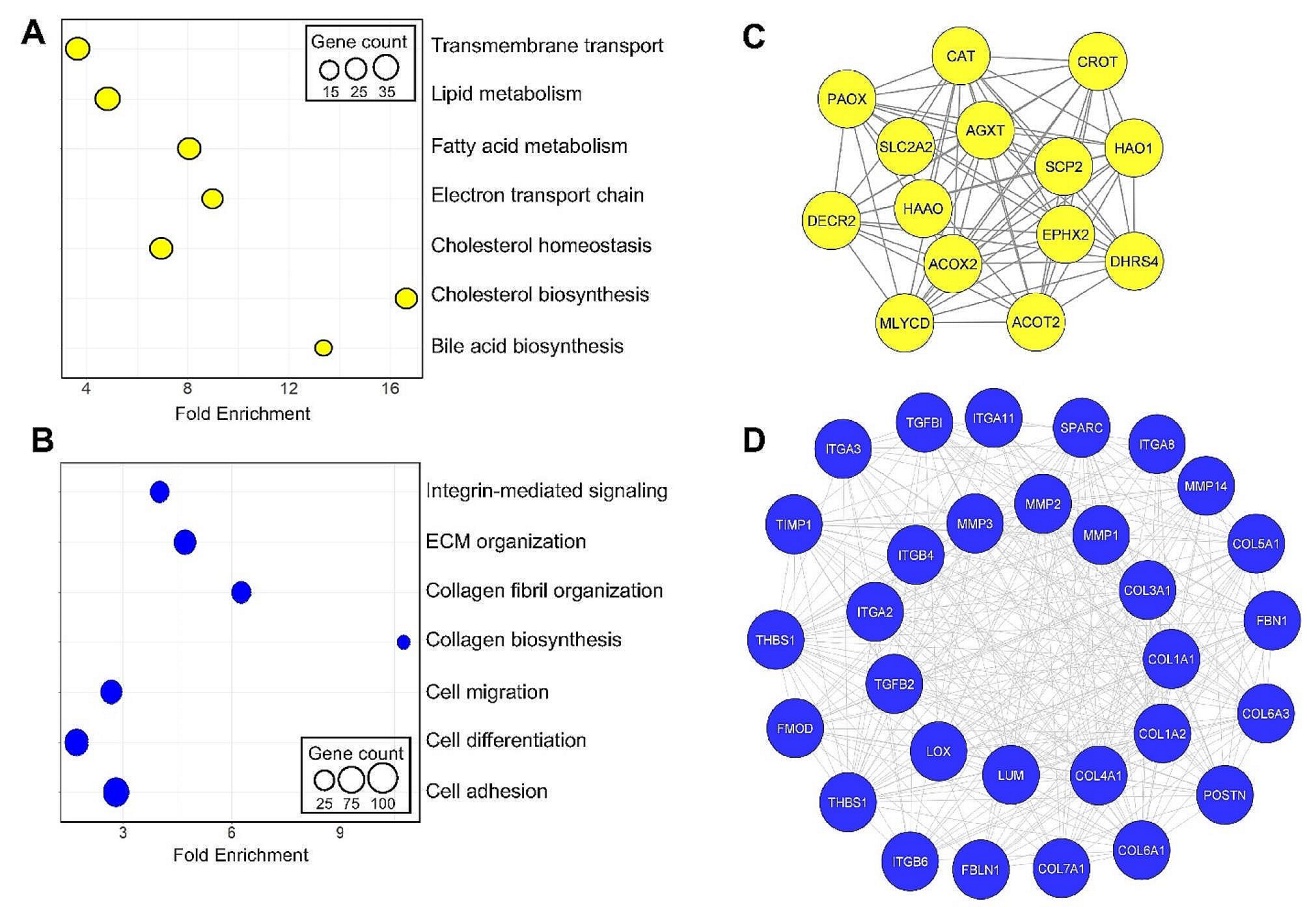
Shared molecular features between a subset of rejected donor livers and the accepted group correspond to metabolic functions and extracellular matrix organization. (**A, B**) The enriched pathways were obtained using the DAVID Functional Gene Annotation tool with a false discovery rate (FDR < 0.05) for common up- and downregulated genes between the accepted and rejected-2 groups. (**C, D**) Protein-protein interaction (PPI) network with the highest interconnectivity for the upregulated genes (**C**) and downregulated genes (**D**) common between the accepted and rejected-2 group compared to the rejected-1 group. The network was generated using the MCODE application in the Cytoscape, with nodes representing the interacting proteins and edges representing the interactions. The highly interconnected network module of the upregulated genes (14 nodes, 71 edges) is enriched for core metabolic functions of the liver (**C**), whereas the network module of the downregulated genes (30 nodes, 310 edges) is enriched for extracellular matrix organization (**D**)

Pathway enrichment analysis revealed that the upregulated genes in accepted and rejected-2 were mainly enriched for lipid metabolism, fatty acid metabolism, cholesterol biosynthesis, cholesterol homeostasis, electron transport chain, bile acid biosynthesis, and transmembrane transport (False Discovery Rate < 5%) (Fig. 4A). The downregulated genes in accepted and rejected-2 were mainly enriched for extracellular liver matrix organization, integrin-mediated signaling, collagen biosynthesis, collagen fibril organization, cell adhesion, cell migration, and cell differentiation (Fig. 4B).

Our analysis thus far is based on comparing the rejected-2 group with the accepted donor livers for similarity of transcriptomics, suggesting similar functional capacities. As an additional comparative analysis for the suitability of transplantation of the rejected-2 group, we considered a published data set on transcriptomically derived classifier genes for predicting successful grant function post-transplantation [27]. The classifier consists of 69 down-regulated genes and 9 up-regulated genes in non-IPGF vs. IPGF. We tested the hypothesis that genes with an expression signature predictive of post-transplantation success are enriched in the rejected-2 subset relative to the rejected-1 group. We found that non-IPGF vs. IPGF classifier genes showed a robust signature in our transcriptomics data, and out of the 69 downregulated genes in the non-IPGF group that showed successful graft function post-transplantation, 47 genes were also downregulated in the rejected-2 and accepted groups compared to the rejected-1 group in our study (Supplementary Fig. 1A, B).

We constructed protein-protein interaction networks for the genes overlapping between the accepted and rejected-2 groups. The most interconnected module of the upregulated genes contained 14 proteins (nodes) and 71 interactions (edges) corresponding to fatty acid metabolism, lipid metabolism, amino acid metabolism, and glucose transport (Fig. 4C). The most interconnected module of the downregulated genes contained 30 proteins and 310 interactions corresponding to ECM organization, cell adhesion, cell-matrix interactions, tissue integrity, and remodeling (Fig. 4D). These findings highlight the extensive overlap in transcriptomic features spanning system-wide liver functions between the accepted and rejected-2 groups of donor livers. In particular, the molecular characterization and pathway enrichment analysis indicated that the high expression of liver metabolic functional genes and low expression of fibrotic genes are likely to be similar in the accepted and rejected-2 donor livers, with implications on the suitability for transplantation of the latter group.

### Integrated Molecular profiling, Histopathological analysis, and Clinical factors predict a cohort of rejected donor livers as potentially suitable for transplantation

**Fig. 5:**
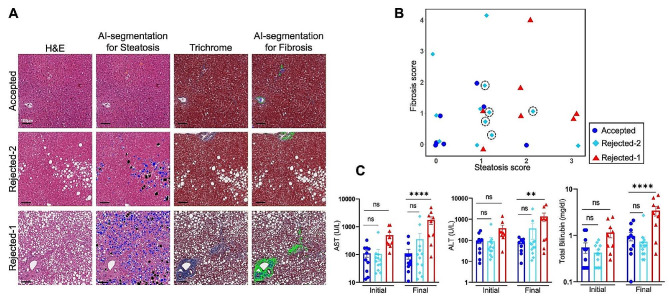
Integrated transcriptomics and histopathological analysis identified a cohort of rejected donor livers potentially suitable for transplantation. (**A**) Histopathological AI-segmented images of liver biopsy tissue sections from the accepted, rejected-1, and rejected-2 groups stained with H&E (left panel) and Trichrome (right panel). Microsteatosis is highlighted in blue, macrosteatosis in black, and fibrosis in green. Scale bar: 100 μm. (**B**) Scatter plot depicting pathologist-assessed histopathology scores of steatosis and fibrosis in donor liver biopsies from the accepted, rejected-1, and rejected-2 groups. The dashed circles indicate the selected donor livers in the rejected-2 group that are potentially suitable for transplantation based on molecular overlap with the accepted group and histopathology evaluation for steatosis, fibrosis, necrosis, and hepatocyte ballooning (Table 2). (**C**) Serum AST, ALT, and total bilirubin levels in the accepted, rejected-1 and rejected-2 groups. Statistical significance was calculated using two-way ANOVA Error bars: mean ± standard error of the mean, two-way ANOVA ****p_adj_<0.0001,**p_adj_<0.01, n.s. p_adj_>0.05

We identified a subset within the rejected group, rejected-2, that is molecularly similar to accepted donor livers. We filtered the rejected-2 subset based on histopathological analysis to assess micro- and macrosteatosis, fibrosis, necrosis, and hepatocyte ballooning (Table 2; Fig. 5). As expected, the accepted group showed no steatosis or minimal steatosis, no fibrosis, or perisinusoidal fibrosis (Fig. 5A, B). In contrast, donor livers within the rejected-2 subset displayed a range of tissue steatosis, varying from no steatosis to 40–70% steatosis. Fibrosis in the rejected-2 donor livers ranged from no fibrosis, perisinusoidal fibrosis, portal, and periportal fibrosis to bridging fibrosis (Fig. 5A, B). Conversely, the rejected-1 subset showed steatosis exceeding 33% in most biopsy samples, with some exhibiting more than 50% steatosis. Furthermore, most liver biopsies in the rejected-1 subset demonstrated fibrosis, ranging from perisinusoidal fibrosis and portal & periportal fibrosis to bridging fibrosis and even cirrhosis. Additionally, some donor livers in the rejected-1 group show high necrosis and hepatocyte ballooning, whereas there was no necrosis in the accepted donor livers (Table 2). There was no necrosis in most of the rejected-2 group except for two donor livers with mild to moderate necrosis. We compared AST, ALT, and total bilirubin levels across the three liver groups. Consistent with the transcriptomics findings, there were no statistical differences between the accepted and rejected-2 groups, whereas there were significant differences in the AST, ALT, and total bilirubin levels compared to the rejected-1 group (Fig. 5C). Histopathological filtering of the molecularly identified rejected-2 subset revealed 5 of the 12 donor livers as potentially transplantable (highlighted in Fig. 5B). These findings highlight the potential utility of incorporating transcriptomic analysis alongside current histopathological selection criteria to aid in decision-making regarding the transplantability of donor livers.

**Table 2:** Percentage of steatosis, fibrosis, necrosis, and hepatocyte ballooning as evaluated by a gastrointestinal pathologist. The percentage of steatosis and tissue collagen were also obtained using AI-based image analysis implemented in the Visiopharm software. The histopathological scores were assigned by the pathologist according to the following criteria – Steatosis: <5% − 0; 5–33% − 1; 34–66% -1; >66% − 3; Fibrosis: None − 0; Mild zone 3 perisinusoidal fibrosis − 1a; Moderate zone 3 perisinusoidal fibrosis − 1b; Portal fibrosis − 1c; Portal and periportal fibrosis − 2; Bridging fibrosis − 3; Cirrhosis – 4; Necrosis: None/minimal − 0; Moderate/Mild − 1; Moderate to severe − 2; Severe – 3; Hepatocyte Ballooning: None − 0; Mild, few − 1; Moderate or marked − 2

UNOS-ID	Group	Steatosis score, %	Steatosis, AI computed	Fibrosis score, description	Collagen, AI computed	Necrosis	Ballooning
AIWL117	Accepted	0, 0%	2%	0, None	3%	0, None	0, None
AHHC458	Accepted	2, 40–50%	29%	0, None	8%	0, None	0, None
AHHQ205	Accepted	0, 0%	1%	1a, Perisinusoidal fibrosis	2%	0, None	0, None
AIBQ043	Accepted	1, 5–10%	4%	2, Portal & periportal	24%	0, None	0, None
AIBF419	Accepted	1, 15%	10%	1b, Perisinusoidal fibrosis	3%	0, None	0, None
AJCN178	Accepted	0, < 5%	4%	0, None	5%	0, None	0, None
AJCT312	Accepted	0, < 5%	3%	0, None	25%	0, None	0, None
AHH5160	Rejected-1	3, 80%	70%	1a, Perisinusoidal fibrosis	5%	0, None	1, Mild, few
AHIE299	Rejected-1	1, 10%	None	0, None	8%	3, Severe	0, None
AHHH143	Rejected-1	2, 50–60%	54%	1a, Perisinusoidal fibrosis	1%	1, Mild	1, Mild, few
AHG2102	Rejected-1	2, 40%	10%	4, Cirrhosis	14%	0, None	2, Moderate
AHKL087	Rejected-1	2, 60%	37%	2, Portal & periportal	2%	0, None	2, Moderate
AHIR183	Rejected-1	3, 80%	64%	1a, Perisinusoidal fibrosis	1%	1, Mild	1, Mild, few
AHGW406	Rejected-1	1, 5%	7%	1a, Perisinusoidal fibrosis	2%	0, None	0, None
AHK2146	Rejected-2	3, 70%	37%	0, None	2%	0, None	1, Mild, few
AHL5250	Rejected-2	0, < 5%	4%	3, Bridging	3%	0, None	0, None
AHKN132	Rejected-2	1, 15%	9%	3–4, Bridging to rare nodules	8%	0, None	1, Mild, few
AHJQ339	Rejected-2	0, None	2%	1a, Perisinusoidal fibrosis	5%	1, Mild	0, None
AHGI142	Rejected-2	1, 15%	13%	1a, Perisinusoidal fibrosis	6%	0, None	2, Moderate
AHGO418	Rejected-2	0, < 5%	4%	0, None	4%	2, Moderate	0, None
AHJB400	Rejected-2	1, 20%	28%	1a, Perisinusoidal fibrosis	4%	1, Mild	0, None
AHII090	Rejected-2	1, 10%	9%	2, Portal & periportal fibrosis	10%	0, None	0, None
AHLH202	Rejected-2	2, 40%	15%	0, None	3%	0, None	2, Moderate
AHLJ351	Rejected-2	2, 40%	42%	1c, Portal fibrosis	2%	0, None	1, Mild, few
AHKC275	Rejected-2	1, 30%	12%	1a, Perisinusoidal fibrosis	2%	0, None	2, Moderate
AHCE041	Rejected-2	1, 30%	32%	0, None	2%	0, None	0, None

## Discussion

**Fig. 6:**
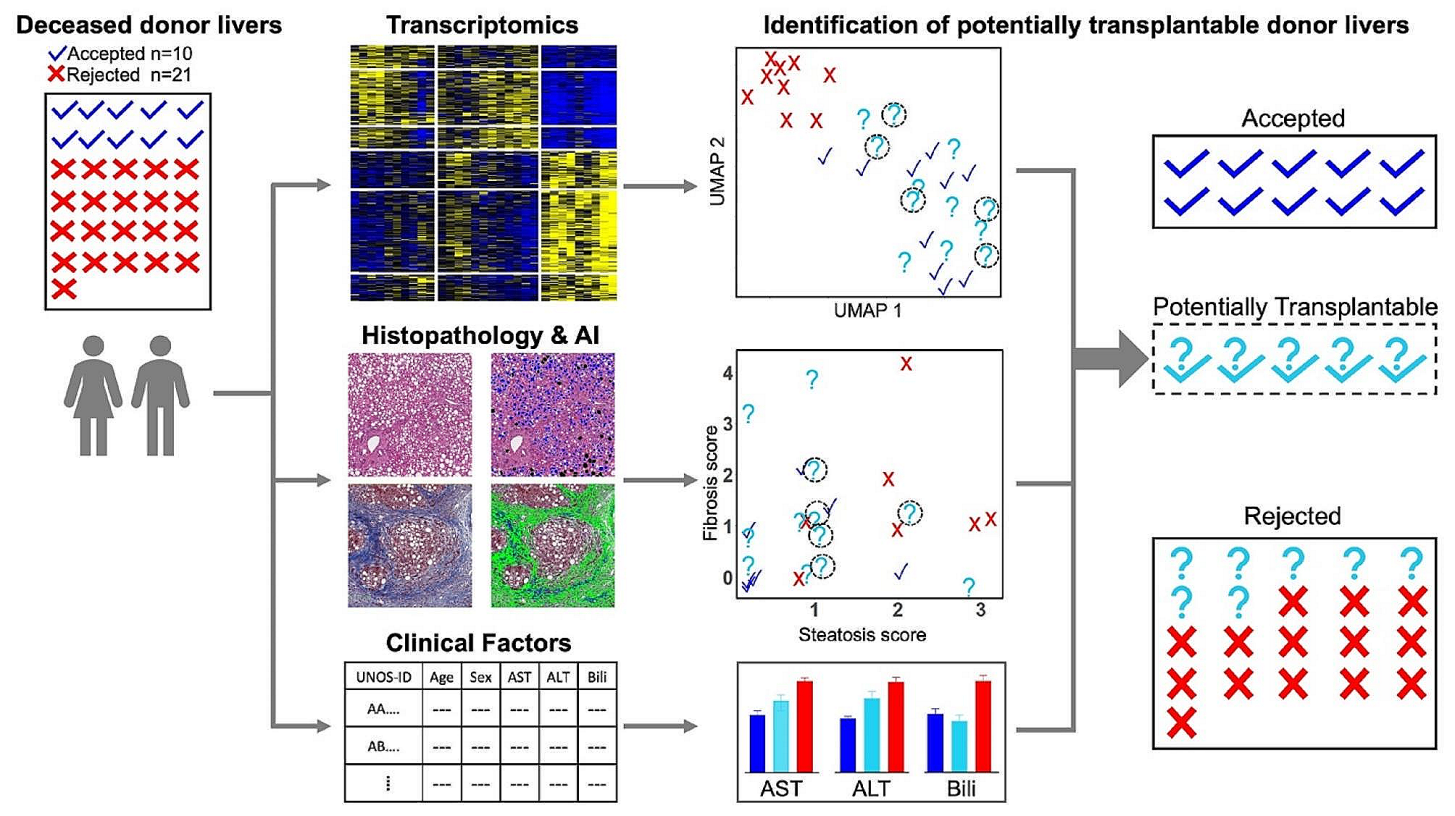
Integrating transcriptomics, histopathology and clinical factors to identify donor livers potentially suitable for transplantation. Transcriptomics identified a subset of rejected livers that had largely overlapping gene expression profiles with those accepted for transplantation. Histopathology analysis allowed filtering of the transcriptomically-derived subset of donor livers further based on for fibrosis, steatosis and necrosis (not shown) levels. The clinical factors informative of liver function were consistent with combined analysis of transcriptomics and histopathology. Together, these complementary approaches yielded a subset of donor livers that are potentially suitable for transplantation

We implemented an integrated transcriptomics and histopathological approach to characterize deceased donor livers that were either accepted or rejected for transplantation (Fig. 6). We performed bulk RNA sequencing to characterize the global gene expression profiles. Additionally, we conducted a histopathological evaluation through manual assessment by a gastrointestinal pathologist and AI-based image analysis. Gene expression analysis revealed high variability within rejected donor livers, with a fraction of rejected donor livers showing extensive transcriptomic overlap with the accepted group. Liver functional tests for serum AST, ALT, and total bilirubin showed similar overlapping patterns. These results suggest functional similarities across critical liver pathophysiological processes related to metabolic functions and ECM organization between some rejected donor livers and the accepted group, pointing to the potential suitability of transplantation of these livers. Importantly, the transcriptomic pattern of this subset of potentially suitable livers was enriched for a gene expression signature of graft success post-transplantation. Additional filtering of the rejected group by histopathological evaluation identified a select subset of donor livers that are likely suitable for transplantation.

Guidelines for assessing the suitability of donor livers for transplantation are lacking, leading to considerable variability among transplant centers [21]. Our approach captures the variability of selection criteria across transplant centers. This variability affects the rejected group as livers considered borderline by one center may be acceptable for transplantation by another. The wide range of gene expression at least partly reflects the variability in the selection criteria that led to rejection for transplantation. The accepted group was less variable in gene expression, likely reflecting the consistency of the selection criteria within a transplant center (TJUH). Together, the transcriptomic consistency of the accepted group and the relatively higher molecular heterogeneity of the rejected group enabled us to identify a subset of livers likely suitable for transplantation based on the molecular state of the donor liver at the time of organ recovery. Our results are further supported by the enrichment of a published transcriptomics signature linked to post-transplantation graft failure or success [27]. Follow-up studies can build on our findings using perfusion and functional testing of livers identified as potentially transplantable through an integrated transcriptomics and histopathological approach.

Biomarker assay development is largely focused on predicting post-liver transplant success [26, 27]. There are no studies on a diagnostic biomarker-based assay to support decision-making in selecting donor livers during organ recovery. Our current transcriptomics data provides a resource to identify such predictive biomarkers. The underlying transcriptomic profiles of the livers typically accepted for transplantation reflect preserved functions crucial for liver homeostasis and show no evidence of tissue damage. Additionally, linking histopathological features to underlying molecular characteristics corresponding to functions critical to liver pathophysiology may help predict the suitability of donor livers solely based on histopathological analysis. Such an approach has the potential to overcome the existing challenges of obtaining molecular profiling data within the critical time frame between organ recovery and transplantation and also serves as a cost-effective alternative to expensive multigene molecular assays. For example, in the case of renal transplantation, a molecular assay based on gene expression signature was developed to complement histopathological evaluation [38]. A combined use of molecular and histopathological evaluation has shown promise in overcoming the considerable variability across centers in evaluating renal transplant suitability of donor kidneys [38–40]. Recent efforts through the VITTAL study demonstrated that a large fraction (71%) of otherwise rejected livers can be transplanted after normothermic perfusion with 100% graft and patient survival for 90 days and significant patient survival over 5 years post-transplantation [41]. These results are consistent with our transcriptomic findings that a significant fraction of currently rejected livers retain a molecular state similar to that of accepted livers, demonstrating the potential for recovering liver functional capacity and leading to successful graft function post-transplantation.

There is an opportunity to build on our transcriptomics results to define molecular states at the time of organ recovery to predict post-transplant outcomes. Including molecular profiling and defining molecular states of the marginal livers can help guide the parameters for the downstream methods of organ transplant. While the marginal livers could expand the pool of transplantable livers, there is a higher chance of primary graft failure (PGF) compared with standard criteria donor (SCD) grafts [42]. Extrapolating from our results on the molecular overlap between accepted and a subset of rejected livers, the transplantation success of some of the marginal livers could potentially be attributed to the similarity of the underlying molecular functionalities of the marginal livers with that of livers typically regarded as acceptable for transplantation. Importantly, this similarity of underlying molecular state may not be readily apparent in the current clinical practice of histopathological assessment of the marginal livers. Biomarker evaluation guided by our transcriptomics results and potential integration with histopathology may improve decision-making in evaluating marginal livers for transplantation. Our study includes both DBD and DCD livers in all sample groups. The rejected-1 and rejected-2 groups included both DCD and DBD cases, demonstrating that our results in subgrouping the rejected donors are not biased by the underlying cause of death. We applied an integrated transcriptomics and histopathological approach and identified the subset within the rejected group. Our data presents an opportunity for leveraging transcriptomic information to discover molecular biomarkers that could play a crucial role in the selection of DCD livers for transplantation.

## Conclusions

The present study utilized an integrated transcriptomic and histopathological approach to characterize transplant donor livers from biopsies of deceased donor livers either accepted or rejected for liver transplantation at the time of organ collection. Our integrated approach led us to identify potentially transplantable livers within the rejected group, which leads to exciting translational opportunities for our findings, pointing to an opportunity to expand the pool of donor livers that could be used in transplantation. Our findings hold the potential to address the variability observed between transplant centers in the selection of donor livers for transplantation, where the current evaluation primarily relies on histopathology, and there is variability in the application of the selection of donor organs for transplantation. In future endeavors, we aim to construct a comprehensive landscape of morphological features in donor liver samples and integrate these findings with our transcriptomics data to strengthen the ability to predict the suitability of the donor liver for transplantation. Moreover, we intend to leverage our integrated approach to predict post-transplant success. Our results demonstrate a proof of principle for the utility of molecular characterization and histopathological assessment in evaluating donor livers for transplantation.

### Electronic supplementary material

Below is the link to the electronic supplementary material.

Supplementary Material 1

Supplementary Material 2

Supplementary Material 3

Supplementary Material 4

Supplementary Material 5

## Data Availability

RNA sequencing data are available on NCBI Gene Expression Omnibus: GEO accession ID GSE243887.

## Abbreviations

ALB: Albumin
ALT: Alanine aminotransferase
APP: Application Protocol Packages
AST: Aminotransferase enzyme
CPM: Counts per million
CYP2E1: Cytochrome P450 Family 2 Subfamily E Member 1
DBD: Donation after brain death
DCD: Donation after circulatory death
ECD: Extended criteria donor
GOT: Glutamic-oxaloacetic transaminase gene
GPT: Glutamate pyruvate transaminase gene
H&E: Hematoxylin & Eosin
MASLD: Metabolic dysfunction-associated steatotic liver disease
MCODE: Molecular Complex Detection
MELD: Model for End-Stage Liver Disease
OPTN: Organ Procurement and Transplantation Network
PFA: Paraformaldehyde
PGF: primary graft failure
PPI: Protein-protein interaction
SCD: Standard criteria donor
SLC2A2: Carrier Family 2 Member 2
TJUH: Thomas Jefferson University Hospital
UMAP: Uniform manifold approximation and projection
WSI: Whole slide images
