# Supplementary material for: Integrated transcriptomics and histopathology approach identifies a subset of rejected donor livers with potential suitability for transplantation
Source: BMC Genomics. 2024 May 2;25:437. doi: 10.1186/s12864-024-10362-7 (PMC11067109; doi:10.1186/s12864-024-10362-7)
Supplement: Supplementary file 1 — Supplementary Material 1 [file 12864_2024_10362_MOESM1_ESM.pdf]

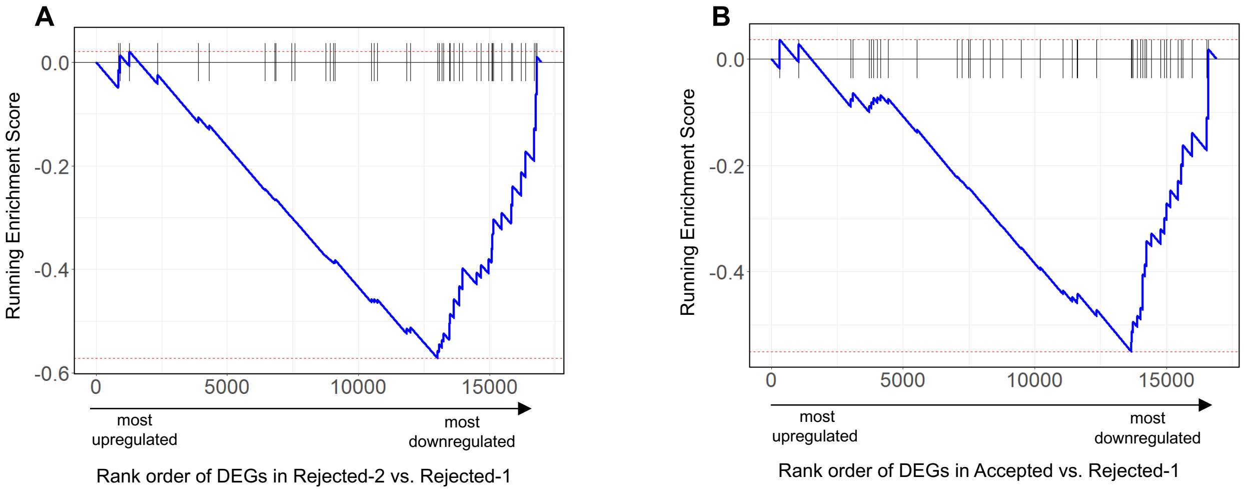

Supplementary Figure 1. Gene Set Enrichment Analysis evaluating the statistical significance of enrichment of a gene expression signature predictive of the graft success post-transplantation. The signature was derived from a published classifier analysis of gene expression changes between livers that showed no initial poor graft function (non-IPGF) vs. IPGF (27). The plots illustrate the enrichment of non-IPGF downregulated genes in (A) rejected-2 donor livers and (B) accepted donor livers when compared to the rejected-1 subset.

Table S1. Number of Transplant centers that considered and rejected the deceased donor livers.

Table S2. The normalized expression values of all genes across all samples.

Table S3. Complete list of genes annotated with differential expression statistics for a comparison between the accepted and the rejected groups, sorted according to the adjusted p-value.

Table S4. Complete list of genes annotated with differential expression statistics for a comparison between the accepted and the rejected subsets (accepted vs. rejected-1, rejected-2 vs. rejected-1, and accepted vs. rejected-2).
