# Supplementary material for: Integrated transcriptomics and histopathology approach identifies a subset of rejected donor livers with potential suitability for transplantation
Source: BMC Genomics. 2024 May 2;25:437. doi: 10.1186/s12864-024-10362-7 (PMC11067109; doi:10.1186/s12864-024-10362-7)
Supplement: Supplementary file 2 — Supplementary Material 2 [file 12864_2024_10362_MOESM2_ESM.pdf]

Supplementary Table S1. Number of Transplant centers that considered and rejected the deceased donor livers.

UNOS-ID	Number of Transplant Centers that rejected the deceased donor livers
AHH5160	33
AHIE299	51
AHHH143	54
AHG2102	20
AHKL087	78
AHIR183	43
AHGW406	12
AIAC389	104
AIAJ296	15
AHK2146	45
AHL5250	46
AHKN132	41
AHJQ339	34
AHGI142	28
AHGO418	38
AHJB400	69
AHII090	107
AHLH202	47
AHLJ351	50
AHKC275	51
AHCE041	31
